# Stratifying Brain Tumour Histological Sub-Types: The Application of ATR-FTIR Serum Spectroscopy in Secondary Care

**DOI:** 10.3390/cancers12071710

**Published:** 2020-06-27

**Authors:** James M. Cameron, Christopher Rinaldi, Holly J. Butler, Mark G Hegarty, Paul M. Brennan, Michael D. Jenkinson, Khaja Syed, Katherine M. Ashton, Timothy P. Dawson, David S. Palmer, Matthew J. Baker

**Affiliations:** 1 WestCHEM, Department of Pure and Applied Chemistry, Technology and Innovation Centre, University of Strathclyde, 99 George St, Glasgow G1 1RD, UK; j.m.cameron@strath.ac.uk (J.M.C.); christopher.rinaldi@strath.ac.uk (C.R.); 2 ClinSpec Diagnostics, University of Strathclyde, Technology and Innovation Centre, 99 George Street, Glasgow G1 1RD, UK; holly.butler@clinspecdx.com (H.J.B.); mark.hegarty@clinspecdx.com (M.G.H.); 3 Translational Neurosurgery, Department of Clinical Neurosciences, Western General Hospital, Edinburgh EH4 2XU, UK; paul.brennan@ed.ac.uk; 4 Institute of Translational Medicine, University of Liverpool & The Walton Centre NHS Foundation Trust, Lower Lane, Fazakerley, Liverpool L9 7LJ, UK; Michael.Jenkinson@liverpool.ac.uk; 5 Walton Research Tissue Bank, Neurosciences Labs, The Walton Centre NHS Foundation Trust, Lower lane, Fazakerley, Liverpool L9 7LJ UK; khaja.syed@thewaltoncentre.nhs.uk; 6 Neuropathology, Lancashire Teaching Hospitals NHS Trust, Royal Preston Hospital, Sharoe Green Lane North, Preston, Lancashire PR2 9HT, UK; katherine.ashton@lthtr.nhs.uk (K.M.A.); timothy.dawson@lthtr.nhs.uk (T.P.D.); 7 WestCHEM, Department of Pure and Applied Chemistry, Thomas Graham Building, University of Strathclyde, 295 Cathedral Street, Glasgow G1 1XL, UK; david.palmer@strath.ac.uk

**Keywords:** Brain Cancer, Infrared, Spectroscopy, Serum, Diagnostics, Tumour Stratification

## Abstract

Patients living with brain tumours have the highest average years of life lost of any cancer, ultimately reducing average life expectancy by 20 years. Diagnosis depends on brain imaging and most often confirmatory tissue biopsy for histology. The majority of patients experience non-specific symptoms, such as headache, and may be reviewed in primary care on multiple occasions before diagnosis is made. Sixty-two per cent of patients are diagnosed on brain imaging performed when they deteriorate and present to the emergency department. Histological diagnosis from invasive surgical biopsy is necessary prior to definitive treatment, because imaging techniques alone have difficulty in distinguishing between several types of brain cancer. However, surgery itself does not necessarily control tumour growth, and risks morbidity for the patient. Due to their similar features on brain scans, glioblastoma, primary central nervous system lymphoma and brain metastases have been known to cause radiological confusion. Non-invasive tests that support stratification of tumour subtype would enhance early personalisation of treatment selection and reduce the delay and risks associated with surgery for many patients. Techniques involving vibrational spectroscopy, such as attenuated total reflection Fourier transform infrared (ATR-FTIR) spectroscopy, have previously demonstrated analytical capabilities for cancer diagnostics. In this study, infrared spectra from 641 blood serum samples obtained from brain cancer and control patients have been collected. Firstly, we highlight the capability of ATR-FTIR to distinguish between healthy controls and brain cancer at sensitivities and specificities above 90%, before defining subtle differences in protein secondary structures between patient groups through Amide I deconvolution. We successfully differentiate several types of brain lesions (glioblastoma, meningioma, primary central nervous system lymphoma and metastasis) with balanced accuracies >80%. A reliable blood serum test capable of stratifying brain tumours in secondary care could potentially avoid surgery and speed up the time to definitive therapy, which would be of great value for both neurologists and patients.

## 1. Introduction 

Brain cancer reduces a patient’s average life expectancy by 20 years on average, the highest reduction of all cancers [1]. Although brain tumours are uncommon, the annual incidence appears to be rising, with an increase of 19% in the United Kingdom (UK) between 2002 and 2014 [2]. Globally, around 330,000 people are diagnosed with a central nervous system (CNS) cancer per year, which equates to ~900 diagnoses every single day [3]. Fewer than 20% of patients survive beyond five years [4], which is considerably lower than other cancer types. 

The current gold standard investigation for patients with a suspected brain tumour is MRI, however determining the exact brain tumour type is not possible from imaging alone [5,6]. Some brain tumours pose particular imaging challenges, e.g. differentiating between glioma and primary CNS lymphoma (PCNSL). Consequently, oncological treatments (radiotherapy and chemotherapy) can only be initiated after histopathological diagnoses are obtained. This necessitates surgery (either resection or biopsy), and although surgery is the primary treatment option for most brain tumours, it is not always clinically indicated or appropriate. This includes patients with borderline performance status who might not benefit from treatment [7]. In patients where a biopsy is only required for histological diagnosis, the time taken to schedule and recover from surgery delays instigation of definitive treatment. 

The detection of brain cancer with a serum-based triage system would be well-suited to the clinical environment. Serum tests are already used in clinics, and a new test could be readily integrated into the current clinical pathway [8]. A rapid blood test that can stratify brain tumour histological sub-type would positively impact on the diagnosis and personalisation of patient treatment. Strategies for non-invasive assessment of tumour type, so-called liquid biopsy, have, to date, largely relied upon identification of cell-free tumour DNA (ctDNA) in circulating blood. This approach has met with significant technical challenges, as well as high cost per test. 

An alternative strategy uses vibrational spectroscopy, in particular attenuated total reflection Fourier transform infrared (ATR-FTIR) spectroscopy, for serum analysis. ATR-FTIR is rapid, cheap and non-invasive, instruments are easy to operate, and the technique generates biochemical fingerprints from minute volumes of biological fluids. In FTIR spectroscopy, a sample is irradiated with infrared light, which causes atomic displacements and molecular vibrations. The absorption of this light excites vibrational transitions of molecules, producing IR spectra that contain a vast amount of chemical and biological information [9]. Specifically, it provides qualitative interrogation of all infrared active macromolecular constituents of blood serum. It has been shown that biomolecular imbalances in biofluids can give an indication of disease states [10]. When coupled with complex data analysis systems, the technique has been shown to successfully detect various cancers [11]. Recently, we have employed this technology in a novel high-throughput approach clinical study, supporting the possibility of earlier detection of brain tumours by identifying which patients with non-specific symptoms of a possible brain tumour are most likely to actually have a tumour, demonstrating high sensitivity and specificity [12]. 

We have used this same FTIR spectroscopy and data analysis strategy to successfully differentiate between two types of brain tumours, glioblastoma (GBM) and PCNSL, which pose a dilemma in radiological diagnosis [13]. If we can differentiate likely tumour types across a broader range of tumour types when an intracranial abnormality is identified radiologically, this would enhance clinical decision making and may reduce the need for some diagnostic investigations, such as body CT in patients with primary brain disease [14]. A simple and reliable blood test that is able to differentiate a range of primary brain tumours from brain metastases would be invaluable to neurologists in the secondary care setting. Thus, in this study we further expand our previous work by assessing various brain tumour subtypes—including meningioma, GBM and PCNSL—and, for the first time, we elucidate the capability of high-throughput ATR-FTIR to differentiate between GBM and brain metastases. The ability to predict the likely diagnosis through a combination of serum spectroscopy and brain imaging would have a major impact on the patient pathway, and would facilitate more timely treatment in the hospital care setting.

## 2. Materials and Methods

### 2.1. Sample Collection and Preparation

A total of 641 retrospective serum samples were obtained from two biobanks; the Walton Centre NHS Trust (Liverpool, UK) and Royal Preston Hospital (Preston, UK). Ethical approval for this study was obtained (Walton Research Bank and BTNW/WRTB 13_01/ BTNW Application #1108). Figure S1 outlines the number of samples within the patient cohort for each category. A respectable balance of male and female patients has been included, with a widespread age range (Table S1). Initially, individual brain tumour types were compared to healthy controls. The larger groups of brain tumour patients were analysed, followed by a breakdown of tumour types. The ‘glioma’ cohort was comprised of the tumours originating from glial cells; GBM, astrocytomas and oligodendrogliomas. The gliomas were contrasted to the meningioma samples, and these two groups were then combined to form the ‘primary’ set, which was tested against the brain metastases. Some of the more abundant individual tumour types were then chosen for further analysis. 

Control patients were healthy individuals who had no history of cancer. The cancer patients had a histopathologically confirmed brain tumour, but had not yet commenced chemo- or radiotherapy at the time of blood sample collection. Blood samples were collected in serum collection tubes and allowed to clot for up to one hour. The tubes were centrifuged for 15 min at 2200× *g*. The serum component was subsequently aliquoted then stored in a –80 °C freezer until the time of analysis. The serum samples were removed from storage and thawed at room temperature (18–25 °C) for approximately 20 minutes prior to spectral analysis. An amount of 3 µL of serum from one individual patient was pipetted onto each of the three sample wells on a ClinSpec Dx optical sample slide (ClinSpec Diagnostics Ltd., UK) [12]. The first well remained clean for background collection to subtract atmospheric conditions from the IR spectra. The serum drops were spread across the well in order to create thin homogeneous serum films. Prepared slides were stored in a Heratherm™ drying unit incubator (Thermo Fisher Scientific, Waltham, MA, USA) at 35 °C for 1 hour to optimise the drying process [15].

### 2.2. Spectral Collection

A Perkin Elmer Spectrum 2 FTIR spectrometer (Perkin Elmer, London, UK) was used for the spectral collection. A ClinSpec Dx indexer (ClinSpec Diagnostics Ltd., Glasgow, UK) automated the movement of the slides across the specular reflectance puck. With the first well acting as a background, the three sample wells provided the biological repeats. Each well was analysed in triplicate—resulting in nine spectra per patient. The spectra were acquired in the range 4000–4500 cm^−1^, at a resolution of 4 cm^−1^, with 1 cm^−1^ data spacing and 16 co-added scans. In total, 5769 spectra have been collected from all serum samples. 

### 2.3. Spectral Analysis

Principal component analysis (PCA) was processed using Matlab, and the PRFFECT toolbox within R Statistical Computing Environment software was utilised for the pre-processing and classifications [16]. Correcting for variation in baselines and using appropriate data reduction methods, such as binning and smoothing, can emphasise valuable biological information—such an approach is known as ‘pre-processing’ [17]. For the PCA, the spectral datasets were cut to the biologically relevant fingerprint region (1800–900 cm^−1^), followed by a rubber band baseline correction and a vector normalisation. PCA is a relatively basic linear transformation technique that is often used in spectroscopic studies. The main aim of a PCA analysis is to identify distinct patterns in complex data and detect a correlation between variables [18]. Ultimately, the dimensionality of large datasets is reduced, in order to clearly visualise the general variation, which can be achieved through scores and loadings plots. 

Curve fitting analysis was carried out on MagicPlot (Magicplot Systems LLC, Saint Petersburg, Russia) in order to unveil the hidden protein secondary structure bands concealed within the broad Amide I region. The mean absorbance and second derivative spectra of the control, GBM, PCNSL, metastasis and meningioma patient groups were processed using the PRFFECT toolbox. A Savitzky–Golay filter was applied to reduce the noise, with the number of smoothing points set to 7. The curve-fitting procedure is based on the sum of Lorentzian functions, which exist at the location of overlapping peaks [19]. Thus, the positions of the minima observed in the second derivative spectra were used to calculate the position and intensity of the Lorentzian curves, which could then be tentatively assigned as various types of protein secondary structures [20]. 

Classifications were undertaken to recognise biological signatures from cohorts of patients with known tumour types, then predictions from ‘unknown’ tumour types were made using this information. Firstly, the spectra were suitably pre-processed. Using a trial-and-error approach, the optimal parameters for the classifications were established. An extended multiplicative signal correction (EMSC) was employed using a human pooled serum reference, followed by a spectral cut to 1800–1000 cm^−1^. A min–max normalisation between 0 and 1, and a binning factor of 8 were applied. To develop the models, patients were randomly split into training sets—consisting of 70% of the data—and test sets—the remaining 30%. Model hyperparameters were tuned to optimise the value of Cohen’s Kappa coefficient (𝜅) for 5-fold cross-validation on the training sets. The optimised model was then used to make predictions for the spectra in the test sets. The majority vote amongst the nine spectra for each patient was reported as the diagnostic outcome. The classification models were retrained and tested on 100 different randomly selected training and test set partitions to provide a reliable measure of predictive accuracy with a low standard error. Due to the imbalances present when examining the different classes, up-sampling, down-sampling and synthetic minority over-sampling technique (SMOTE) were employed in the spectral analysis to reduce the bias in the classification models. Three robust classification techniques have been employed in this study: random forest (RF), partial least squares-discriminant analysis (PLS-DA), and support vector machine (SVM). For a thorough explanation of each of these methods and their parameters, we direct the reader to our previous work [13]. Briefly, RF builds a ‘forest’ of regression trees using the Classification and Regression Trees algorithm [21], and by applying the Gini impurity metric, it can rank spectral features in order of importance. PLS-DA is similar to PCA, in that it can extract hidden patterns from complex datasets by reducing the dimensionality [22]. The supervised SVM technique can output an optimal dimension for the separation of the data, known as the hyperplane. Various kernels are available, but here we use a linear kernel, which has previously been shown to perform well in spectral classification studies [23].

## 3. Results

### 3.1. Brain Tumour vs. Healthy Control

#### 3.1.1. Principal Component Analysis

PCA was first undertaken in order to explore the general variation between the controls and the individual brain tumour groups. The data was cut to the fingerprint region where biomolecules are known to vibrate (1800–900 cm^−1^), before a rubberband baseline correction and vector normalisation was applied. Firstly, the GBM patients were compared to the healthy individuals. Figure S2 describes: a) the scores plot between PC1 and PC2 for GBM against controls (NC), and b) and c) are the loadings plot for PC1 and PC2, respectively. PC1 accounts for 52.3% of the general variation in the dataset, mainly from Amide I and II contributions, as shown in Figure S2b). Despite some slight overlap, the two groups separate across the 2nd principal component. The PC2 loadings also suggest that this arises from the Amide I (CO and CN stretch, NH bending) and Amide II (NH bending, CN stretch) bands between 1500–1700 cm^−1^. There were also contributions from the lower wavenumber region, which represents the CO, CC and CH stretching modes from carbohydrates and glycogen, and the symmetric ${\mathrm{PO}}_{2}^{-}$ stretching vibrations within nucleic acids (1100–1000 cm^−1^). 

Meningioma, PCNSL and metastatic patient cohorts were also assessed individually against the control group. Figure S3 displays the PCA results for each of the comparisons. The scores plots and loadings presented are those that illustrate the most discrimination between classes. Similar to GBM, with the meningioma and PCNSL analysis, we found that the PC2 suggested that the most variance arose at the Amide I and II bands and at the phosphate, glycogen and carbohydrate region. Interestingly, the region around 1080–1000 cm^−1^ was shown to exhibit the highest discrepancies in the metastasis vs. control set. The ~1030 cm^−1^ band is associated with the CO stretching and bending vibrations of glycogen and carbohydrates. This is consistent with a previous study, where this region was found to be distinctive when analysing normal and metastatic brain tumour tissue through FTIR imaging and linear discriminant analysis [24].

#### 3.1.2. Amide I Deconvolution

**Figure 1:**
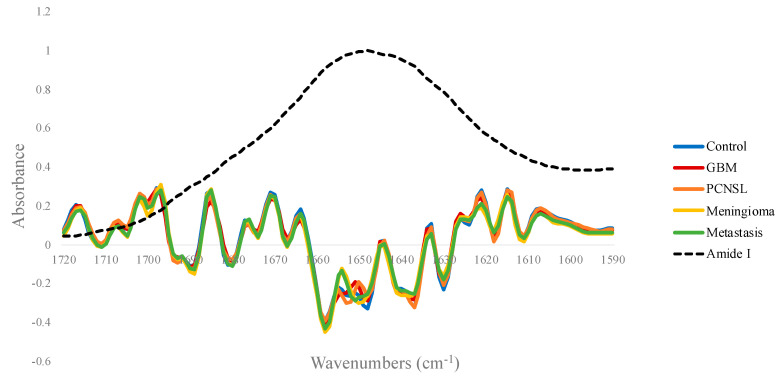
The mean second derivative spectra within the Amide I region (1720–1590 cm^−1^) for the control, glioblastoma (GBM), primary central nervous system lymphoma (PCNSL), meningioma and metastasis patient groups.

The PCA analysis highlighted variances in Amide I absorbance between brain tumour groups, thus deconvolution analysis was undertaken to further explore these differences. A series of overlapping components that represent different structural elements are hidden within the broad Amide I band [25,26]. For example, β-sheets involve two or more segments of a polypeptide chain lining up next to each other and form a sheet-like structure, as the C=O of one amino acid binds to the N-H of another through hydrogen bonding, whereas α-helices are assembled when the polypeptide chain twists into a spiral [27]. For the four brain tumour subtype groups and the control set, the mean Amide I absorbance spectra were subjected to a second derivative deconvolution in an attempt to better understand the nature of the identified spectral differences. Figure 1 describes the second derivative spectra in the region between 1720–1590 cm^−1^ for each patient set, which suggests that there are minute discrepancies at several points across the Amide I band, namely at ~1650 cm^−1^ and ~1638 cm^−1^. 

**Figure 2:**
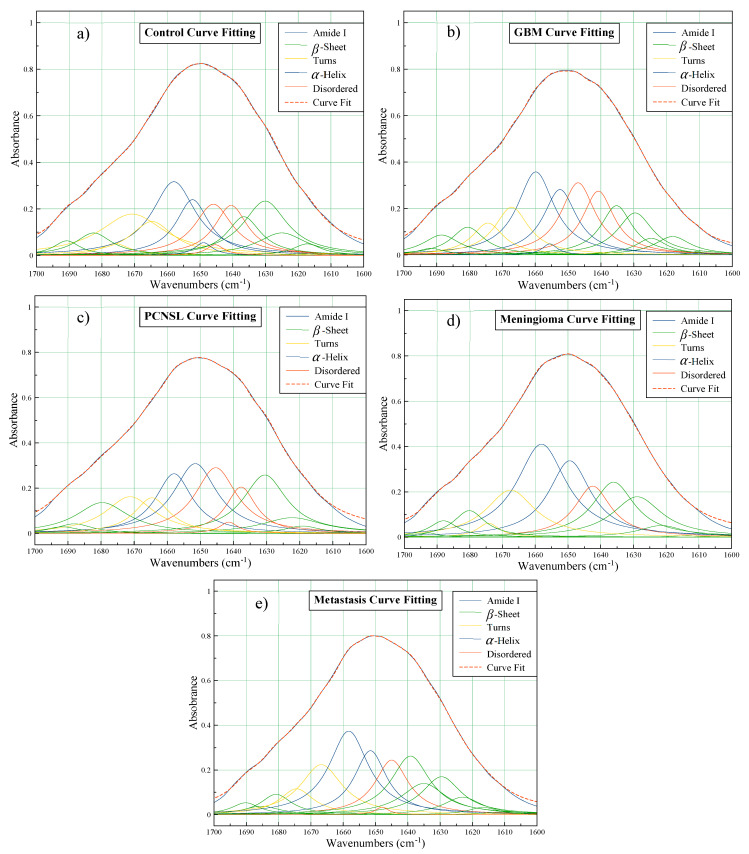
Amide I curve fitting showing the summation of resolved second derivative bands relative to the absorption profile for the: (**a**) control; (**b**) glioblastoma (GBM); (**c**) primary central nervous system lymphoma (PCNSL); (**d**) meningioma and (**e**) metastasis patient groups.

The position and intensity of the minima across the second derivative spectra represent those of the underlying protein bands, so it is possible to predict secondary structures using curve fitting. The deconvoluted Amide I profiles for each of the patient groups are distinctly dissimilar, in terms of the number of bands, and their relative positions and intensities. The curve fitting analysis is outlined in Figure 2, where the overlapping protein bands have been tentatively assigned as either α-helices, β-sheets, turns or random disordered structures with reference to the literature [26,28]. 

Initially, it seems that all of the deconvoluted Amide peaks follow a similar trend. From left to right, β-sheets exist around 1700–1680 cm^−1^; followed by turns ~1670 cm^−1^; then the elevated α-helix bands and disordered structures between 1665 and 1645 cm^−1^; finally, additional β-sheet components from 1640 to 1600 cm^−1^. On closer inspection, it is clear that the profiles are rather disparate. Despite all patient groups consistently encompassing α-helix maxima, they all exist at fluctuating heights and positions. Interestingly, this region exhibited discrepancies in the second derivative spectra in Figure 1, corroborating the differences observed in the curve fitting analysis. 

#### 3.1.3. Partial Least Squares-Discriminant Analysis

Based on our previous work [13], we used PLS-DA to classify each dataset and test the diagnostic performance. Initially, each model was tested with no additional sampling, before using up, down and SMOTE sampling techniques to ensure there was no bias present within the classifications, which could be introduced by the imbalanced classes. The optimal value of *ncomp* for each model was determined from a tuning grid with a range 1:20 (Table S2). The sampling method that produced the best results with five iterations was then chosen for 100 resamples, to generate the most accurate and optimal outcome. Table 1 outlines the PLS-DA results for each tumour type vs. control dataset.

The analysed GBM vs. control set contained 96 GBM patients and 87 controls, hence the sampling techniques—for equalising imbalanced patient groups—did not significantly improve the classification results. After 100 iterations, the PLS-DA model reported 95.5% and 94.9% for sensitivity and specificity, respectively. The SDs were minimal for both sensitivity and specificity (~4%), suggesting the model is robust and reproducible. Likewise, the ability to successfully pick out the PCNSL, meningioma and metastatic patients from their respective training sets was also evident. The number of patients in these groups were not well matched, thus additional up sampling seemed to improve the performance of the models. The sensitivities after 100 resamples were 92.2% for PCNSL, 94.7% for meningioma and 95.9% for metastasis. The tests were also highly specific, with each model accurately predicting the healthy controls as non-cancer at specificities ≥ 95%. 

**Figure 3:**
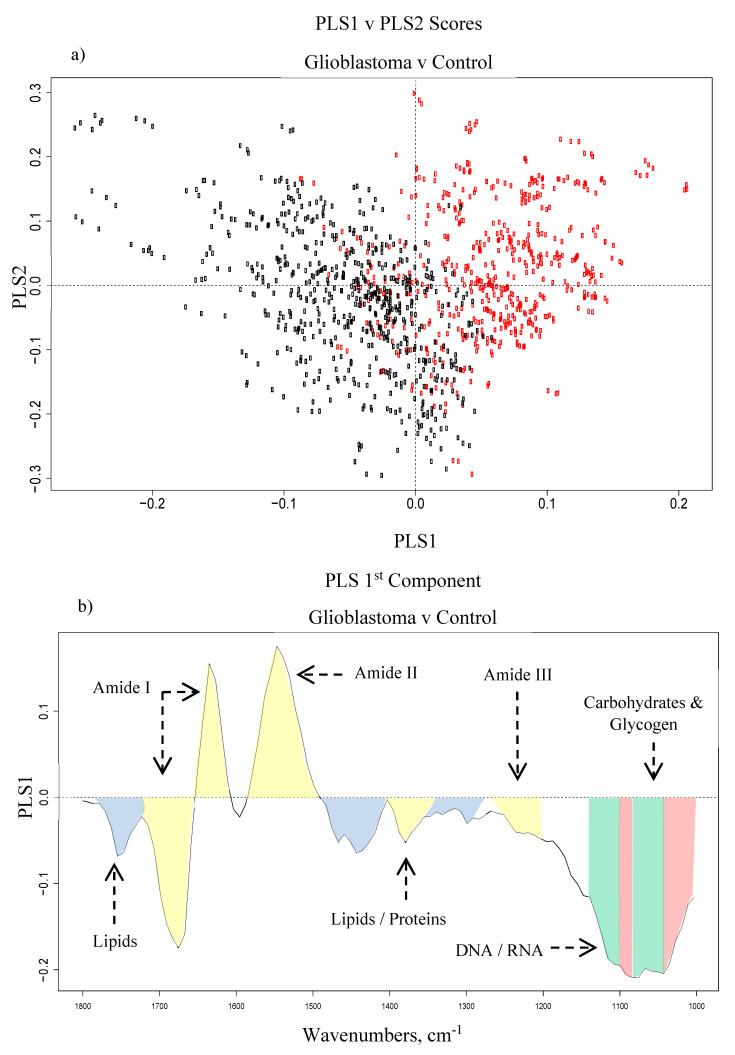
(**a**) Partial least squares (PLS) scores plot between PLS1 and PLS2 for the glioblastoma (black) and control (red) dataset, and (**b**) the loadings for the 1st PLS component with tentative biological assignments: lipids (blue), proteins (yellow), phosphates (green) and carbohydrates (red).

The PLS scores plots were very similar to the PCA results, but they provided slightly better separation of the classes. Figure 3 shows (a) the PLS scores plot between PLS1 and PLS2, and (b) the loadings for the 1st PLS component based on the GBM vs. control dataset. The PLS1 loadings in Figure 3b) generally agree with the PCA loadings (Figure S2). The most discriminating regions in each of the four brain tumour subtypes vs. control datasets were generally found between 1000–1100 cm^−1^ and 1500–1700 cm^−1^, along with some minor lipidic contributions. The wavenumbers that were mainly responsible for all four classifications are outlined in Table 2 with their corresponding biological assignments and vibrational modes. 

Overall, the classification results highlight the ability of ATR-FTIR to successfully differentiate individual brain tumour types from control serum samples with extremely high accuracies. A recent health economic assessment of current diagnostic practices suggested that a serum-based test for the detection of brain tumours could be cost-effective to the NHS [29]. Thus, the results from this retrospective analysis indicate that this platform technology may be well suited to the clinical environment. Moreover, the Amide I deconvolution analysis has highlighted concealed differences in the proteinaceous structures of the different brain tumour types, suggesting that using similar classification techniques, it may also be possible to discriminate between brain lesions as well as brain tumour vs. control.

### 3.2. Brain Tumour Differentiation

We next examined the ability of ATR-FTIR spectroscopy to distinguish the various brain tumour subtypes from each other, rather than individual brain tumour subtypes from controls. We built on our previously reported method to differentiate GBM and PCNSL [13], where RF, PLS-DA and linear SVM were utilised and compared. The SMOTE, up and down sampling techniques were tested to combat the imbalanced classes, and the best model for each classification (Table S2) was iterated 100 times for more reliable results. The optimum model is reported for each combination, in terms of sensitivity, specificity and balanced accuracy, with their corresponding SD (Table 3). In each instance, the sensitivity refers to the positive class and the specificity refers to the negative class. For example, in the glioma vs. meningioma classifier, the sensitivity relates to glioma and the specificity is based on the meningioma predictions.

The classification with the largest number of patients was the primary brain tumour (*n* = 303) vs. brain metastasis (*n* = 210). The best model that was chosen for 100 resamples was the RF with additional up sampling, which provided a sensitivity of 90.9%. This model was evidently very capable of detecting the primary brain tumours within the test set, and on average only missed ~9 out of 90 patients in the resampled test sets. On the other hand, the RF model struggled to detect the metastatic brain tumours in this patient cohort, reporting a rather low mean specificity of 66.4%.

The Gini impurity metric was examined to identify the most important features within each dataset. The accuracy and reliability of the model can be determined from the RF statistical value outputs, with the Gini plot highlighting the main wavenumbers responsible for the results (Figure S4). Table S3 gives an overview of the top 15 identified wavenumbers in order of importance, with their corresponding wavenumber assignments and vibrational modes. As with the brain metastasis vs. control results, the top two Gini values come from the lower wavenumber region around ~1050 cm^−1^, which was found to show the most discrimination between the metastatic and primary tumour samples. The phosphate and CO stretching vibrations from nucleic material and phospholipids give rise to the bands in this region. Stretching of the carbonyl groups in proteins and lipids make up the rest of the top five wavenumbers. These areas of importance are closely followed by Amide I/II/III and lipidic vibrations, as well as contributions from glycogen and carbohydrates.

The optimal results for glioma (*n* = 191) vs. meningioma (*n* = 111) were produced from a linear SVM with down sampling, where random selections of the glioma set were removed from the resampled training sets to have more evenly balanced classes. Down sampling has been criticised in the field for ‘ignoring’ potentially important information, but we overcome this by resampling the data as different random subsets of patients are removed in each iteration. Using this particular method, the SVM model was better at predicting the meningioma patients than picking out the gliomas, reporting a mean sensitivity of 70.2% and a mean specificity of 81.7%. A range of tumour grades are comprised within the glioma group, with lower grade tumours including grade I pilocytic astrocytoma, grade II astrocytomas and oligodendrogliomas, and the higher-grade gliomas dominated by GBMs (grade IV). On average ~16 of the 57 glioma samples in the test sets were misdiagnosed as meningioma, equivalent to a sensitivity of 70%. When the pilocytic astrocytomas, grade II astrocytomas and oligodendrogliomas were removed in order to focus on GBM vs. meningioma, the classification performance was greatly improved, with the sensitivity increasing to 94.4%.

One of the classifications that is of particular interest to clinicians is metastasis vs. GBM. Tumours that transpire to be primary GBMs on histopathology can be confused radiologically with brain metastases [14]. For the resampled SVM model, the sensitivity (metastasis) was 84.3%, and the ability to detect GBM (specificity in this case) was 96.2%. Likewise, using PLS-DA, metastatic patients were separated from PCNSL and meningioma patients with mean balanced accuracies of 91.3% and 78.7%, respectively. Intriguingly, the lesser performance of the metastasis vs. meningioma model was not wholly unexpected. From the second derivative spectra and curve fitting analysis (Figure 1 and Figure 2), it was noticed that their spectral signatures were relatively similar, hence a challenging classification was anticipated.

**Figure 4:**
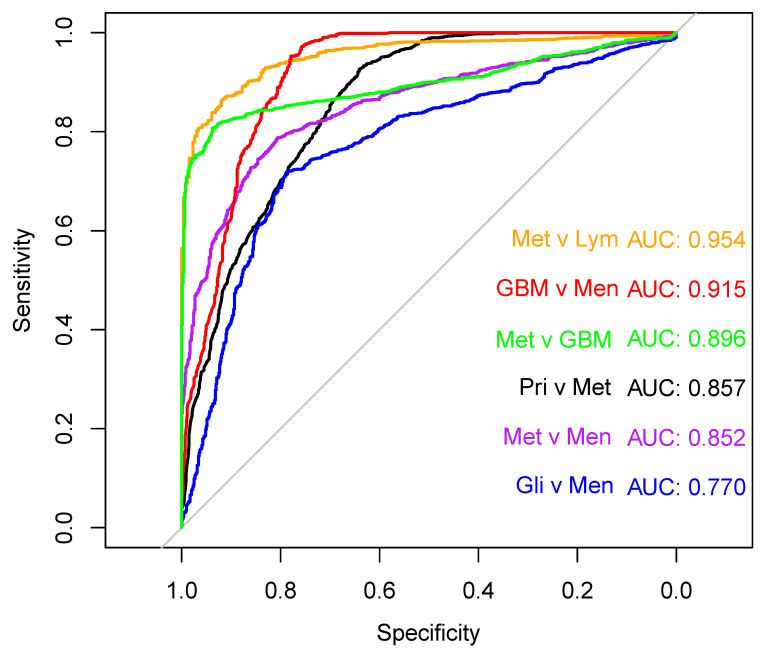
Receiver operator curves displaying the trade-off between sensitivity and specificity for the best model of each of the six brain tumour classifiers: primary (Pri) vs. metastasis (Met), black; glioma (Gli) vs. meningioma (Men), blue; glioblastoma (GBM) vs. meningioma, red; metastasis vs. GBM, green; metastasis vs. primary central nervous system lymphoma (Lym), orange; metastasis vs. meningioma, purple.

The receiver operating characteristic (ROC) curves for each of the brain tumour differentiation models are outlined in Figure 4. The six models have varying diagnostic ability. The GBM vs. meningioma, and the metastasis vs. PCNSL PLS-DA models produce excellent ROC curves, achieving AUC values >0.9. The metastasis vs. GBM linear-SVM model is also highly promising, reporting an AUC of 0.896. Furthermore, the large cohort of primary vs. metastasis and the metastasis vs. meningioma have AUC values ~0.85. The glioma vs. meningioma group yielded the poorest diagnostic capability, with the lowest AUC of 0.77. The AUC values coincide with the classification results in Table 3. Analysis of the ROC curves suggests that some of the presented models could be optimised for clinical applications. A default probability threshold value of 0.5 was used here to distinguish between brain tumour types. However, by varying the probability threshold that each classifier uses to discriminate between positive and negative classes, each model could be fine-tuned to obtain the greatest balance between sensitivity and specificity.

## 4. Discussion

The PCA results described differences between each patient group in the Amide region, which can be attributed to alterations in the levels of proteins. Many proteins exist as circulating markers of inflammation and angiogenesis. For example, C-reactive protein (CRP) and vascular endothelial growth factor (VEGF) were previously reported to be elevated in the plasma of GBM patients [30]. Likewise, various studies have highlighted serum YKL-40 as a potential blood-based biomarker for gliomas, with levels significantly higher in GBM patients in some cases [31,32]. However, there are currently no protein-based biomarkers used for brain tumour differentiation and a signature approach as described here enables a full protein assay to be performed. Separation in PCA score plots was less marked for the other tumour groups than the GBM vs. control analysis. The chemokines, cytokines and other biomarkers that are associated with cancer exist in pg/mL concentrations in serum, and are obscured by larger protein molecules that are present in high concentrations in both cancer and control patients [33,34]. More robust supervised classification techniques are typically required to identify the most salient features within such complex datasets. That being said, PCA offers an unsupervised platform that can indicate specific regions of interest.

Through deconvolution of the Amide I bands, differences in the assignment of certain structures were observed between patient groups. The levels of β-sheets are higher in the PCNSL group when compared to the controls, as well as exhibiting a minor drop-off in α-helices. This is consistent with a previous study, which discriminated lymphoma and normal serum from mouse models [35]. In contrast, there is a decrease in the β-sheet band (~1630 cm^−1^) in the GBM patient group compared to the controls, plus a minor increase in α-helix intensity (~1660 cm^−1^). Interestingly, the PLS1 loadings corroborate these differences (Figure 3b); which defines the variation between the two classes where the GBM patients are the negative cluster and the controls are the positive group, as shown in the scores plot between PLS1 and PLS2 (Figure 3a). When considering the control set, the higher level of β-sheets is described by the intense positive loading at ~1630 cm^−1^, whilst the minor increase in α-helix intensity is observed in the large negative loading around 1660 cm^−1^. Similar findings have been observed recently in a study that utilised synchrotron-based IR micro-spectroscopy to analyse human gliomas, and which demonstrated a rise in the α-helix content while the content of β-sheets decreased with increasing malignancy grade [36]. For the meningioma and metastasis groups, the second derivative spectra were somewhat overlaid (Figure 1), and their deconvoluted bands also seemed to exhibit some noticeable similarities—the intensities of the four largest bands followed the same pattern: two high intensity α-helices at ~1658 and ~1650 cm^−1^, the disordered structure at ~1645 cm^−1^ and a β-sheet at ~1637 cm^−1^, with a similar intensity of ~0.25 on the absorbance scale (Figure 2d,e).

The alterations in protein secondary structures between the mean spectra of respective patient cohorts reflect major biochemical differences in serum content associated with each tumour group. However, blood serum is a complex medium comprised of over 20,000 proteins, which naturally fluctuates between individuals [37]. Hence, the assumption that protein content is irrefutably consistent within patient groups is a slight generalisation. Nevertheless, the technique offers a further insight into the potential variances between the patient groups that have been highlighted through the loadings from PCA and PLS analysis. Furthermore, deconvolution analysis is sensitive to the pre-processing and second derivative parameters that are applied, and indeed these were consistent for this analysis and there are clear differences between tumour types.

It is well recognised the systemic response of cancer impacts the patients’ spectral signatures evident in IR spectroscopy [38,39]. In the case of primary brain tumour vs. metastasis (Figure S4), it may be that the blood composition of the metastasis patients differs slightly from those with brain primaries. One plausible theory is that the levels of cell-free circulating tumour DNA and RNA (ctRNA), and circulating microRNAs (miRNA) are elevated in the bloodstream as a result of the systemic cancer, which could account for the increase in nucleic acid-related absorbance in their spectral serum profile [40,41,42,43,44]. This particular test is of great interest, as if it was possible to tell at an early stage whether a suspected brain tumour was more likely to be a brain primary or a metastatic secondary lesion, it would be both cost- and time-effective for the health services with primary brain tumour patients not requiring further diagnostic body imaging. There are a variety of different metastatic brain tumours arising from different primary cancers (e.g. breast, lung, etc.) within this population. It could be that certain types of lesions have more spectral similarities than others, thus breaking the cohort down into subgroups may benefit classification performance. That said, a balanced accuracy of 78.8% is respectable, and with more thorough tuning of the classification models and by modifying the probability threshold, the sensitivity and specificity could potentially balance out. Moreover, the accuracy could potentially improve with a larger population of metastatic patients.

Likewise, it is unclear exactly why the other glioma types were assigned to the meningioma class, though it could potentially be due to them having a lower growth potential and mitotic activity. This may be reflected by the systemic response to tumour grade, which could influence the respective spectral profiles. As many of the oligodendrogliomas, astrocytomas and meningiomas range between grade I and III, their spectroscopic signature may be more alike than the more aggressive grade IV lesion of GBM.

## 5. Conclusions

In this study, we have assessed serum from patients with various brain tumours, by comparing and contrasting their spectral signatures against each other, as well as healthy controls. GBM, PCNSL, meningioma and brain metastases have been successfully separated from control patients through PLS-DA, all with sensitivities and specificities greater than 92%. Deconvolution of their respective mean Amide I bands highlighted subtle variations in the levels of various protein secondary structures within each tumour type. Hence, further classifications between the lesion classes were fulfilled, presenting some very encouraging results. Despite a relatively low specificity, the primary vs. metastasis cohort showed some initial promise, with the RF model being able to pick out 90.9% of the ‘primary’ brain tumour samples within the resampled test sets. Most other classifiers performed remarkably well for the brain tumour differentiations, achieving balanced accuracies around 80%. Notably, the metastasis vs. GBM linear-SVM classifier reported an 84.3% sensitivity, a 96.2% specificity and a ROC curve with an AUC value of ~0.9, suggesting that the model has high diagnostic capability. Due to their similar features on MRI scans, implementing serum spectroscopy alongside imaging protocols could help differentiate brain metastases from GBM, as well as other tumours with similar radiological appearances, e.g., PCNSL [13,14,45].

A simple and reliable blood test that is able to differentiate a range of primary brain tumour types from brain metastases, would lead to a paradigm shift in the clinical management of brain tumour patients. Our findings in this study suggest this is feasible, and by using basic serum spectroscopic analysis—despite the fact that some of our sample sets had relatively low numbers of patients—all of the presented models achieve balanced accuracies greater than 75% (Figure S5). The ability to provide the likely diagnosis based on a blood test, when combined with radiological assessment, would have a major impact on the patient pathway and would facilitate more timely treatment in the hospital care setting.

For these proof-of-concept tests to be validated, the models must be used to predict tumour type in prospective patients already within the current diagnostic pathway, although these results indicate the potential for a serum diagnostic tool at both the primary and hospital care stage. A reliable blood test in primary care would initially fast-track patients who are in urgent need of referral and brain imaging, whilst reassuring those who have a negative test result and reducing the number of patients who would normally be sent for unnecessary brain scans. Likewise, stratification of brain tumour type through serum spectroscopy would assist clinicians when brain scans are inconclusive and the primary tumour type is uncertain, and furthermore would prevent patients from undergoing avoidable surgical biopsy and/or further MRI and CT imaging. The results of our study show great potential to improve the diagnostic pathway for patients with brain tumours.

## Figures and Tables

**Table 1:** Summary of partial least squares-discriminant analysis (PLS-DA) results for brain tumours against controls. Sensitivity, specificity and balanced accuracy are reported as means and standard deviations (SD) calculated over 100 resamples.

Tumour Type Against Healthy Control (*n* = 87)	No. of Patients	Sampling	Sensitivity (%)		Specificity (%)		Balanced Accuracy (%)	
			Mean	SD	Mean	SD	Mean	SD
GBM	96	No	95.5	4.3	94.9	4.2	95.2	2.9
PCNSL	41	Up	92.2	6.9	96.7	3.5	94.4	3.9
Meningioma	111	Up	94.7	3.7	98.4	2.2	96.6	2.0
Metastasis	210	Up	95.9	2.6	95.0	4.2	95.4	2.3

**Table 2:** The main wavenumbers involved in each of the four brain tumour subtypes vs. control classifications, with tentative biological assignments.

Approximate Wavenumbers (cm^−1^)	Tentative Biological Assignments	Vibrational Modes
1012	Carbohydrate	C-O stretch
1030	Glycogen	C-O and C-C stretch, C-OH deformation
1045	DNA and RNA	symmetric ${\mathrm{PO}}_{2}^{-}$ stretch
1050	Carbohydrate/Glycogen	C-O-C stretching and bending
1050–1100	DNA and RNA	Symmetric ${\mathrm{PO}}_{2}^{-}$ stretch
1240–1310	Amide III of Proteins	N-H in plane bend, C-N stretch
1245	Phosphodiesters	Asymmetric ${\mathrm{PO}}_{2}^{-}$ stretch
1340	Phospholipids	CH_2_ wagging
1400	Lipids/Proteins	CH_3_ bending
1470	Lipids	CH_2_ scissoring
1500–1600	Amide II of Proteins	N-H bending, C-N stretching
1600–1700	Amide I of Proteins	C=O and C-N stretch, N-H bending
1750	Lipids	C=O stretching

**Table 3:** The results from the optimal model for each brain tumour differentiation. Sensitivity, specificity and balanced accuracy are reported as means and standard deviations calculated over 100 resamples.

Classification (Positive Class v Negative Class)	No. of Patients (Positive Class/ Negative Class)	Model + Sampling	Sensitivity (%)		Specificity (%)		Balanced Accuracy (%)	
			Mean	SD	Mean	SD	Mean	SD
Primary v Metastasis	303/210	RF + up	90.9	3.1	66.4	5.5	78.8	2.8
Glioma v Meningioma	192/111	SVM + down	70.9	5.5	81.8	6.2	76.3	4.4
GBM v Meningioma	96/111	RF + no	94.4	5.1	83.4	5.6	88.9	3.0
Metastasis v GBM	210/96	SVM + down	84.3	3.8	96.2	3.4	90.3	2.6
Metastasis v PCNSL	210/41	PLS-DA + smote	91.5	3.1	91.1	9.2	91.3	4.6
Metastasis v Meningioma	210/111	PLS-DA + up	71.3	6.2	86.1	5.5	78.7	3.6

## Supplementary Materials

The following are available online at https://www.mdpi.com/2072-6694/12/7/1710/s1, Figure S1: Breakdown of the large brain cancer cohort with the number of patient samples used for the classifications, Figure S2: (a) Principal component analysis scores plot of PC1 and PC2 displaying the variance between GBM (blue) and healthy control (red); (b) PC1 loadings and (c) PC2 loadings describe which wavenumbers account for the most discrimination, Figure S3: Principal component analysis scores plots displaying the biggest separation between: healthy control (red) versus; (a) meningioma (green), (c) lymphoma (blue) and (e) metastasis (bowel: orange rings, breast: pink rings, lung: green rings, melanoma: blue rings). Corresponding loadings plots for the principle component that describes which wavenumbers account for the separation of; (b) meningioma, (d) lymphoma and (f) metastasis against control, Figure S4: Gini plot outlining the most important features for the Random Forest classification between primary (Pri) and metastasis (Met), Figure S5: Bar graph of balanced accuracies for the differentiation of brain tumour types with their associated standard deviations, Table S1: Age and sex information for each of the tested patient groups, Table S2: Additional information on the classification tuning parameters, Table S3: The top 15 wavenumbers from the Random Forest classification between primary and metastasis with tentative biochemical assignments. The column “ΣGini” is a summation of the mean decrease in Gini for each wavenumber, over all nodes in all trees in the random forest ensemble, which suggests the regions of highest importance.
