# Supplementary material for: Stratifying Brain Tumour Histological Sub-Types: The Application of ATR-FTIR Serum Spectroscopy in Secondary Care
Source: Cancers (Basel). 2020 Jun 27;12(7):1710. doi: 10.3390/cancers12071710 (PMC7408619; doi:10.3390/cancers12071710)
Supplement: Supplementary file 1 [file cancers-12-01710-s001.pdf]

Supplementary Materials: Stratifying Brain Tumour Histological Sub-Types: The Application of ATR-FTIR Serum Spectroscopy in Secondary Care

James M. Cameron, Christopher Rinaldi, Holly J. Butler, Mark G Hegarty, Paul M. Brennan, Michael D. Jenkinson, Khaja Syed, Katherine M. Ashton, Timothy P. Dawson, David S. Palmer and Matthew J. Baker

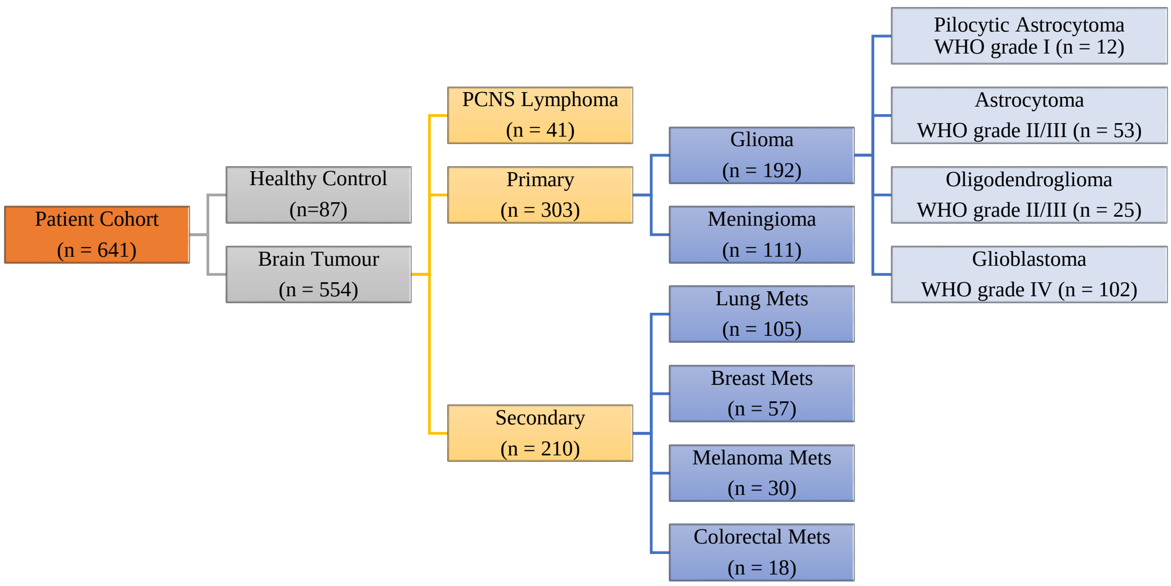

Figure S1. - Breakdown of the large brain cancer cohort with the number of patient samples used for the classifications.

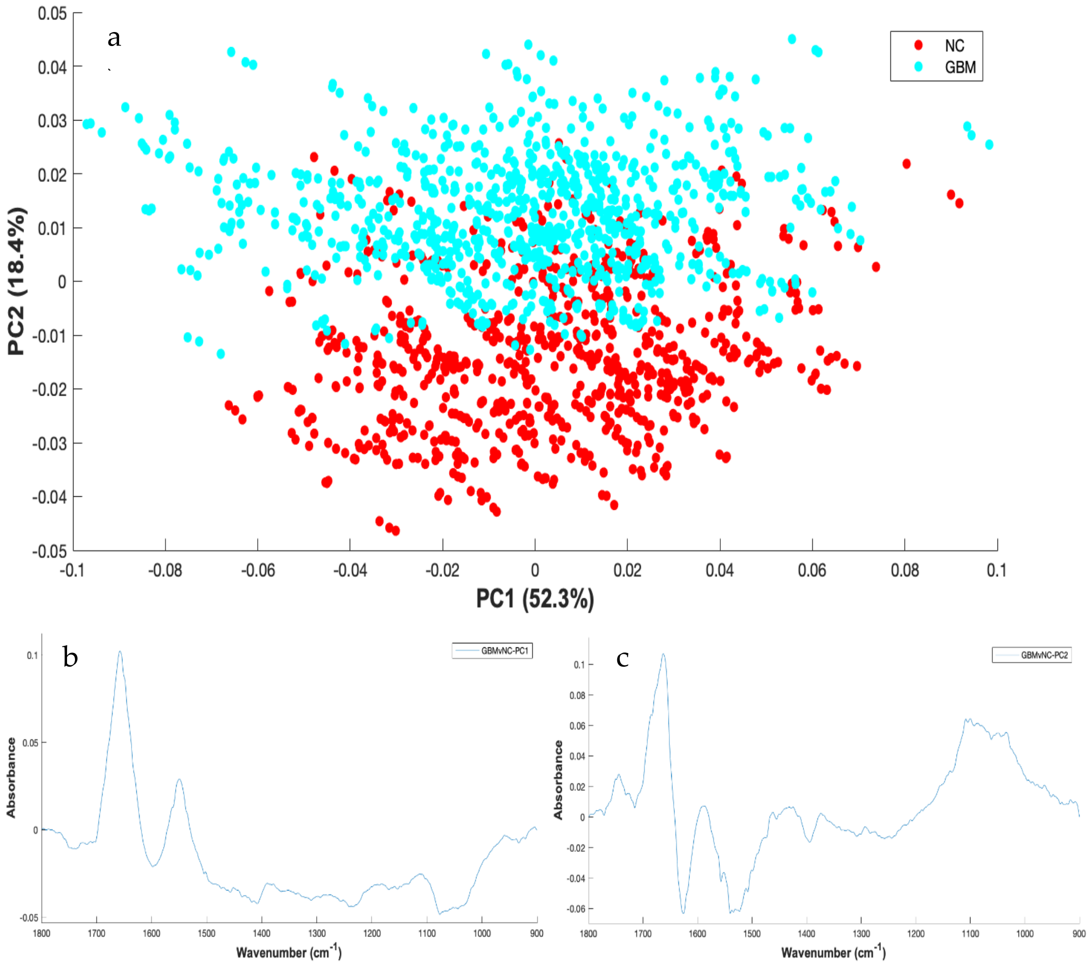

Figure S2. (**a**) Principal component analysis scores plot of PC1 and PC2 displaying the variance between GBM (blue) and healthy control (red); (**b**) PC1 loadings and c) PC2 loadings describe which wavenumbers account for the most discrimination.

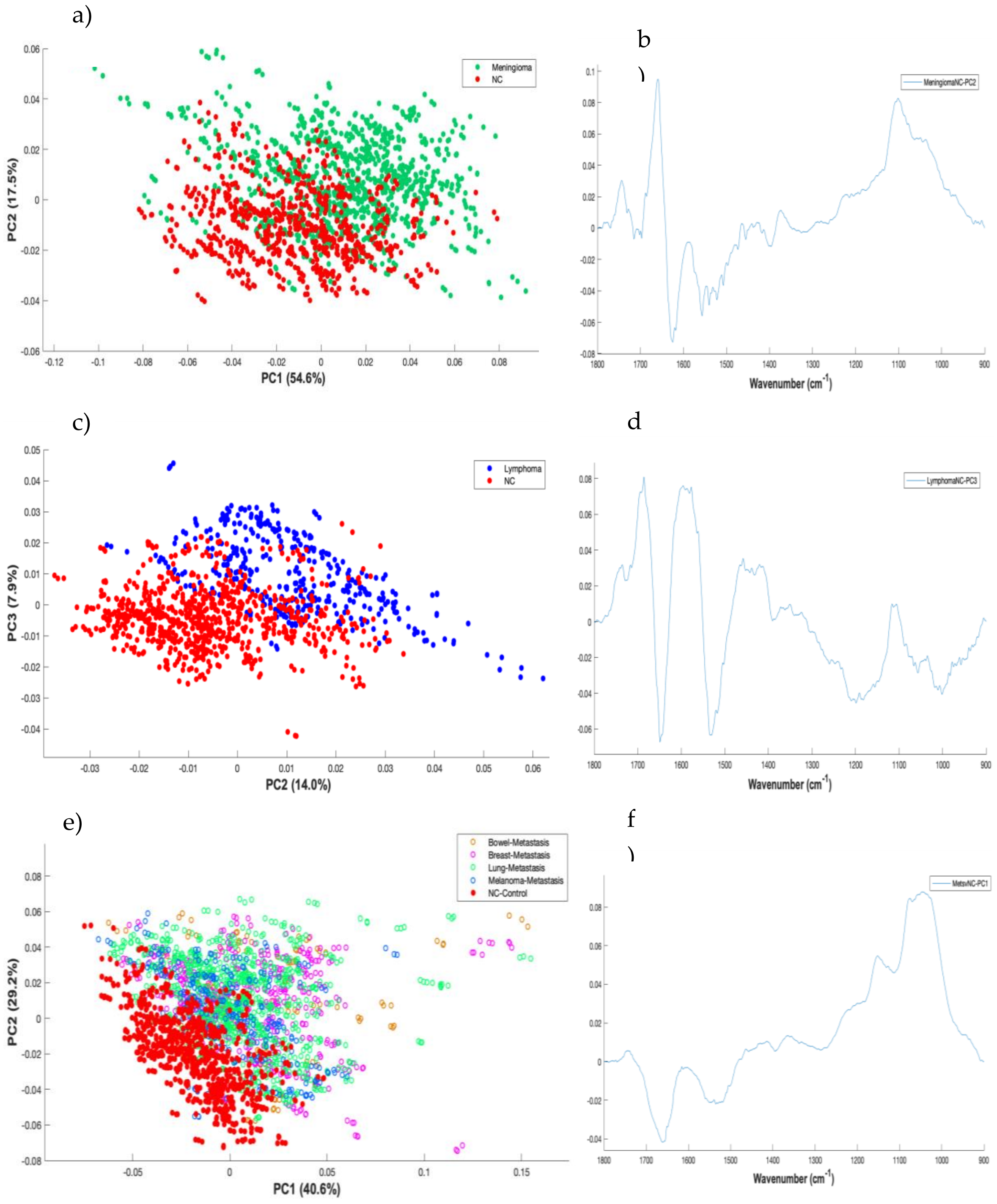

Figure S3. – Principal component analysis scores plots displaying the biggest separation between: healthy control (red) *versus*; **a**) meningioma (green), **c**) lymphoma (blue) and **e**) metastasis (bowel: orange rings, breast: pink rings, lung: green rings, melanoma: blue rings). Corresponding loadings plots for the principle component that describes which wavenumbers account for the separation of; **b**) meningioma, **d**) lymphoma and **f**) metastasis against control.

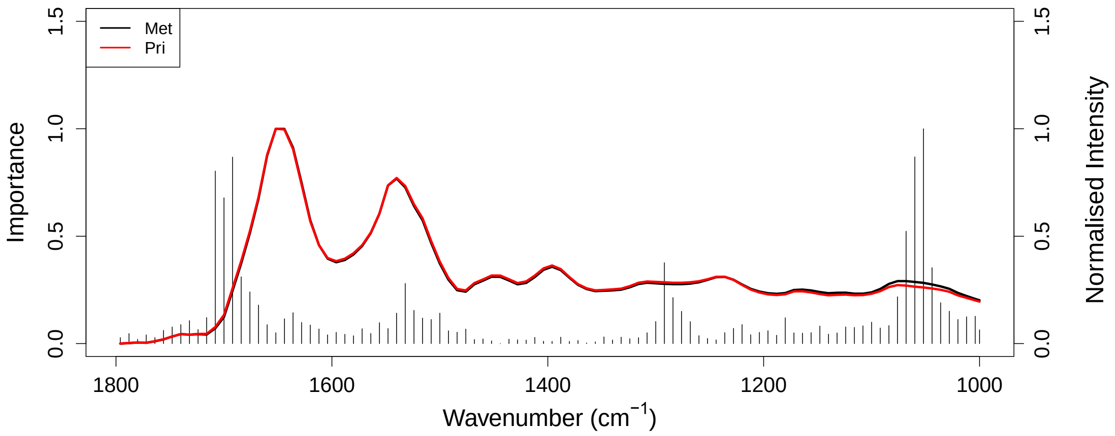

Figure S4. - Gini plot outlining the most important features for the Random Forest classification between primary (Pri) and metastasis (Met).

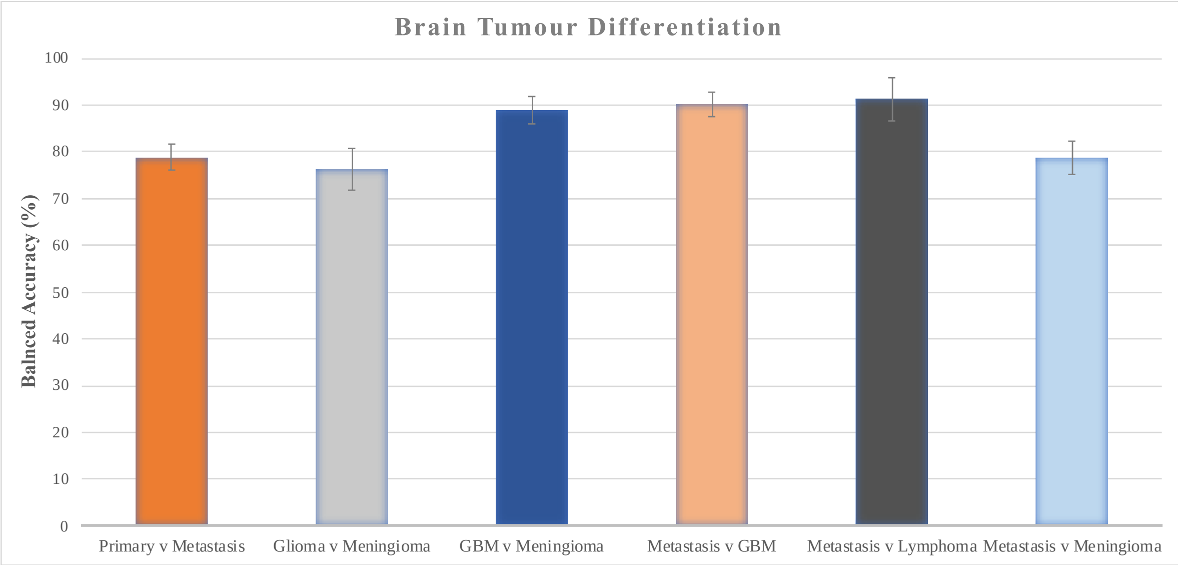

Figure S5. - Bar graph of balanced accuracies for the differentiation of brain tumour types with their associated standard deviations.

Table S1. – Age and sex information for each of the tested patient groups.

	Control	*Primary*	*Glioma*	*PCNSL*	*GBM*	*Meningioma*	*Metastasis*
**Total**	87	303	192	41	102	111	210
**Sex (M/F)**	39/48	163/140	123/69	27/14	62/40	40/71	84/126
**Age Range**	20-64	17-85	17-85	27-86	30-85	26-81	30-86
**Average Age**	35	54	53	60	61	56	61

Table S2. – Additional information on the classification tuning parameters.

**Classification (positive class v negative class)**	*Tuning Parameters*	*Model + Sampling*
**GBM v Control**	***ncomp 10***	PLS-DA + no
PCNSL v Control	*ncomp 14*	PLS-DA + up
Meningioma v Control	*ncomp 16*	PLS-DA + up

Metastasis v Control	*ncomp 13*	PLS-DA + up
Primary v Metastasis	*ntree 500, nodesize 1, mtry 30*	RF + up
Glioma v Meningioma	*cost 0.019*	SVM + down
GBM v Meningioma	*ntree 500, nodesize 1, mtry 30*	RF + no
Metastasis v GBM	*cost 0.019*	SVM + down
Metastasis v PCNSL	*ncomp 10*	PLS-DA + smote
Metastasis v Meningioma	*ncomp 14*	PLS-DA + up

Table S3. The top 15 wavenumbers from the Random Forest classification between primary and metastasis with tentative biochemical assignments. The column "ΣGini" is a summation of the mean decrease in Gini for each wavenumber, over all nodes in all trees in the random forest ensemble, which suggests the regions of highest importance.

Wavenumbers (cm^{-1})	*Σ Gini*	*Tentative biological Assignments*	*Vibrational Modes*
1052.5	123.47	DNA and RNA	Symmetric PO_2^- stretch
1060.5	107.86	Deoxyribose	C-O stretch
1692.5	107.74	Amide I of Proteins	C=O and C-N stretch, N-H bending
1708.5	99.96	Lipids/Fatty acid esters	C=O stretch
1700.5	85.15	Guanine/Thymine	C=O stretch
1068.5	66.56	Ribose/Nucleic acids	C-O stretch
1292.5	49.11	Amide III of Proteins	N-H in plane bend, C-N stretch
1044.5	46.36	Carbohydrate	C-O-C stretch and bending
1684.5	41.31	Amide I of Proteins	C=O and C-N stretch, N-H bending
1532.5	37.61	Amide II of Proteins	N-H bending, C-N stretching
1676.5	32.92	Amide I of Proteins	C=O and C-N stretch, N-H bending
1076.5	30.19	DNA and RNA	Symmetric PO_2^- stretch
1284.5	29.73	Phosphodiesters	Asymmetric PO_2^- stretch
1036.5	26.92	Carbohydrate/Glycogen	C-O and C-C stretch, C-OH deformation
1668.5	25.63	Amide I of Proteins	C=O and C-N stretch, N-H bending
